# Circular RNA as a New Vaccine Platform: Considerations, Challenges, and Perspectives

**DOI:** 10.3390/vaccines14030221

**Published:** 2026-02-28

**Authors:** Kyung Hyun Lee, Jaejin Lee, Seong-Wook Lee

**Affiliations:** 1 R&D Center, Rznomics Inc., Seongnam 13486, Republic of Korea; jjlee@rznomics.com; 2 Department of Bioconvergence Engineering, Research Institute of Advanced Omics, Dankook University, Yongin 16890, Republic of Korea

**Keywords:** RNA vaccine, circular RNA, prophylactic vaccine, preventive vaccine, cancer vaccine, self-circularization, cap-independent translation

## Abstract

Circular RNA (circRNA) has emerged as an alternative RNA modality for vaccine development due to its covalently closed structure and enhanced molecular stability compared with linear messenger RNA (mRNA). Following the clinical success of mRNA vaccines, circRNA-based platforms have gained attention in both prophylactic and cancer immunization. Unlike linear mRNA, circRNA relies on cap-independent translation and is commonly produced through in vitro transcription coupled with ribozyme-mediated self-circularization. In prophylactic vaccination, circRNA vaccines have demonstrated sustained antigen availability, robust humoral and cellular immune responses, and flexibility in multivalent designs and targeted delivery strategies that support germinal center reactions and neutralizing antibody generation. In cancer vaccines, circRNA has been applied to tumor-associated antigens, neoantigens, and non-canonical peptides, with a primary focus on inducing potent antigen-specific CD8^+^ T cell immunity and enabling combination immunotherapy approaches. This review summarizes recent applications of circRNA-based vaccines in prophylactic and cancer settings, emphasizing in vitro transcription-compatible self-circularization strategies and discussing how methodological choices in RNA design, translation control, purification, and delivery shape immunological outcomes.

## 1. Introduction

### 1.1. mRNA Vaccine

Coronavirus disease 2019 (COVID-19), caused by severe acute respiratory syndrome coronavirus 2 (SARS-CoV-2), has emerged as a major global public emergency, posing unprecedented challenges to healthcare systems and societies worldwide. The urgent need for effective countermeasures against COVID-19 accelerated the development of novel vaccine technologies, ultimately leading to the authorization and widespread deployment of mRNA vaccines [1].

The clinical success of mRNA vaccines in controlling the COVID-19 pandemic not only demonstrated their efficacy and safety but also highlighted the advantages of RNA-based vaccine platforms [2]. Unlike conventional vaccines that rely on pathogen cultivation, inactivation, or protein subunit production, mRNA vaccines can be designed once the genetic sequence of a target antigen is available. This feature enables swift responses to emerging infectious diseases and viral variants, underscoring the flexibility of mRNA vaccine development [3].

Importantly, the applicability of RNA-based vaccine platforms is not limited to prophylactic vaccination against infectious diseases [4]. Linear mRNA vaccines have also shown considerable potential in therapeutic settings, particularly in cancer immunotherapy [5]. By enabling endogenous antigen expression within host cells, mRNA vaccines promote antigen processing and presentation through major histocompatibility complex pathways, thereby inducing both robust humoral and cellular immune responses [3]. This dual immune activation supports both effective prophylactic vaccination and the generation of tumor-specific cytotoxic T cell responses in cancer vaccine applications [6].

In addition, the modular and programmable nature of mRNA vaccines allows precise control over antigen sequence, structure, and expression, facilitating rapid adaptation to diverse disease targets [7]. The same manufacturing framework can be readily applied to a wide range of antigens, supporting the use of mRNA vaccines as a versatile and scalable platform across multiple indications, including infectious diseases and cancer.

Despite these advantages, several intrinsic limitations of linear mRNA vaccines have become increasingly apparent. Linear mRNA molecules are inherently unstable and susceptible to exonuclease-mediated degradation, necessitating extensive chemical modifications and sophisticated delivery systems to achieve sufficient stability and translational efficiency [3]. Moreover, stringent cold-chain requirements remain a significant logistical barrier to global vaccine distribution, particularly in resource-limited settings [3].

Another major challenge associated with linear mRNA vaccines is the need to carefully manage innate immune activation [8]. While the incorporation of modified nucleosides has become a standard approach to attenuate excessive immune sensing and enhance translational efficiency [9], this strategy introduces additional practical considerations. In particular, key modified nucleosides and their applications are protected by intellectual property rights, which may limit access for other developers and constrain broader participation in mRNA vaccine innovation. In addition, a recent study suggests that the incorporation of N^1^-methylpseudouridine (m1ψ) can increase ribosomal frame-shifting and generate unintended translation products, underscoring the need for careful sequence-level evaluation even in optimized mRNA designs [10]. Moreover, reliance on extensive nucleoside modification and formulation optimization adds further complexity to vaccine design and development [3].

Collectively, these considerations highlight the need for next-generation RNA vaccine platforms that can maintain the translational and immunological advantages of mRNA vaccines while offering improved molecular stability and more favorable innate immune profiles. In this context, alternative RNA modalities such as circular RNA have emerged as promising candidates [4,11].

### 1.2. Circular RNA Platform

Circular RNA is a class of single-stranded RNA molecules characterized by a covalently closed loop structure that lacks free 5′ and 3′ ends, thereby fundamentally distinguishing it from conventional linear mRNA. Owing to this circular topology, circRNA does not require a canonical 5′ cap or 3′ poly(A) tail and exhibits enhanced stability compared with linear RNA [12]. These features have positioned circRNA as a promising next-generation RNA platform that may preserve the programmability and rapid redesign advantages of RNA vaccines while addressing key limitations associated with linear mRNA [7].

Although circRNA can, in principle, be generated through multiple strategies, vaccine development imposes stringent requirements for scalability, reproducibility, and cell-free manufacturing [3]. Consequently, circRNA vaccine candidates are predominantly produced using in vitro transcription (IVT)-based workflows [13]. In most IVT-based approaches, a linear RNA precursor is first synthesized by IVT, enabling precise control over antigen-coding sequences and regulatory elements, and is subsequently converted into a circular RNA molecule through a dedicated circularization step. The choice of circularization strategy is therefore a critical determinant of product purity, sequence composition at the ligation junction, and downstream translational and immunological properties [11].

**Figure 1:**
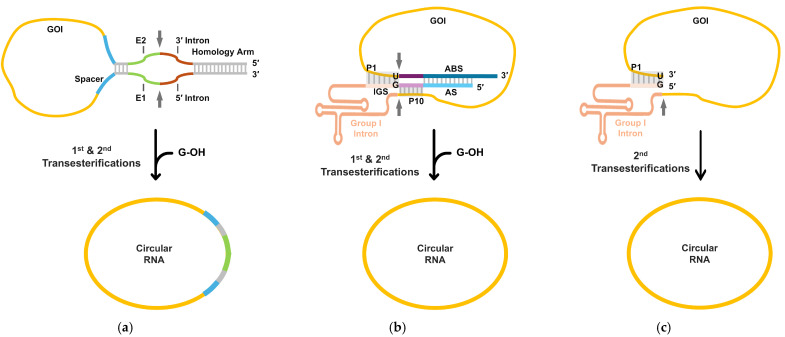
Ribozyme-based in vitro self-circularization methods for circRNA generation: (**a**) PIE-based self-circularization method. In the PIE method, circularly permuted forms of group I intron ribozyme interrupted by an exon can fold into an active conformation. GOI, flanked by E1/E2, intron halves, spacer, and homology arms, undergoes the 1st and 2nd transesterification initiated by exogenous guanosine (G-OH), generating circRNA. After two transesterification reactions, a circular exon with the E1/E2 intronic scar can be generated. For Clean-PIE or SCAP, scarless circRNA can be generated by eliminating E1/E2 by concealing functional splice site-like elements within the GOI. (**b**) STS-based self-circularization method. P1 construct generates scarless circRNA during IVT, intramolecular autocatalytic reaction (two consecutive transesterifications) mediated by a group I intron ribozyme. IGS pairs with the target site in the GOI to form a P1 helix, while P10 defines the splice junctions. G-OH initiates the 1st transesterification, followed by the 2nd transesterification that directly joins the 5′ and 3′ ends of the GOI. (**c**) In the end-to end STS method using the P1 construct, scarless circRNA during IVT can be generated by only the 2nd transesterification. GOI, gene of interest; IGS, internal guide sequence; AS, antisense sequence; ABS, antisense-binding sequence.

Among IVT-compatible approaches, ribozyme-mediated self-circularization has emerged as a particularly practical and widely explored strategy (Table 1), as it enables RNA circularization through autocatalytic reactions without reliance on specific enzymes such as ligases (Figure 1) [13,14]. The permuted intron–exon (PIE) method, based on group I intron ribozymes, has been extensively used [15] and engineered to improve circularization efficiency for longer transcripts (Figure 1a) [16]. Nevertheless, conventional PIE designs generally leave extraneous intronic scar or spacer-derived sequences at the ligation junction [11], which can constrain construct design flexibility and have been suggested to influence innate immune recognition [17].

To mitigate these limitations, several refined self-circularization strategies have been developed. The Clean-PIE or split-CVB3 Ana-PIE (SCAP) methods seek to eliminate extraneous sequences by concealing functional splice site–like elements within the gene of interest (GOI) through rational sequence or codon optimization [18,19]. In parallel, group II intron-based PIE systems have been introduced as alternative ribozyme platforms, enabling scarless circularization through engineered exon–intron recognition mechanisms [20]. Alongside PIE-based approaches, self-targeting and splicing (STS)-based strategies using group I intron ribozymes have been developed, in which circRNA is generated through an intramolecular, end-to-end autocatalytic reaction that directly joins the 5′ and 3′ ends of the GOI (Figure 1b,c) [21]. Such designs enable scarless circularization and can proceed efficiently during the IVT process itself, further underscoring the suitability of ribozyme-mediated self-circularization for scalable circRNA manufacturing. Taken together, these advances highlight self-circularization strategies as a central methodological framework for evaluating and comparing circRNA vaccine platforms, particularly with respect to translational behavior, immunogenicity, and practical manufacturability [11].

Collectively, these features underscore ribozyme-mediated self-circularization as a distinct and advanced IVT-compatible strategy for circRNA production, particularly well-suited for prophylactic/cancer vaccine development and other translational applications [14].

In this review, we summarize recent examples of circular RNA-based platforms applied to prophylactic and cancer vaccines and discuss their design principles, translational characteristics, and remaining challenges toward broader clinical application.

## 2. Circular RNA for Prophylactic Vaccine

**Figure 2:**
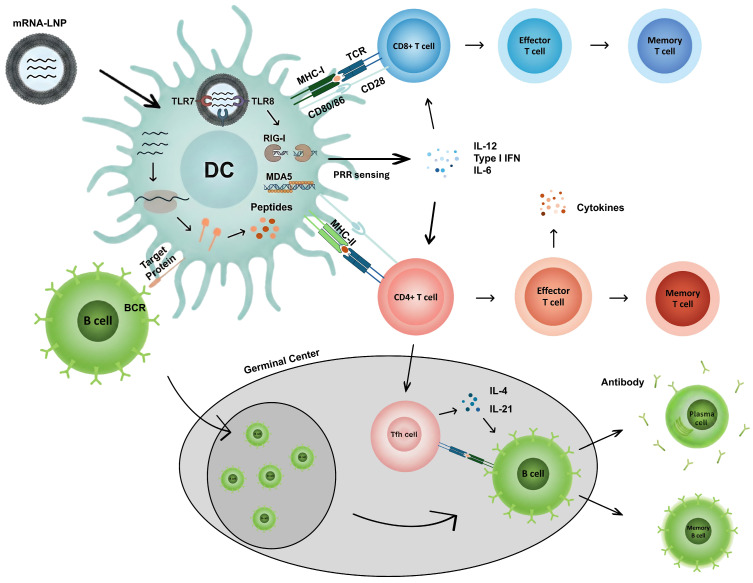
Prophylactic mRNA–LNP vaccine-induced adaptive immune responses. Following administration, mRNA–LNPs are taken up by multiple cell types, including antigen-presenting cells such as dendritic cells (DCs), leading to intracellular antigen expression. In DCs, the mRNA cargo can be sensed by endosomal TLR7/8 and cytosolic receptors such as RIG-I and MDA5, promoting maturation and cytokine production that supports T cell priming through pattern-recognition receptor (PRR) signaling. Antigen-derived peptides are presented on MHC class I and II to prime CD8^+^ and CD4^+^ T cells through TCR recognition with co-stimulatory signals (e.g., CD80/86–CD28), generating effector and memory T cell responses. In particular, IL-12 and type I interferons (IFNs) can contribute to CD8^+^ T cell activation and differentiation. In parallel, antigen availability within lymphoid tissues enables B cell receptor (BCR)-mediated antigen recognition and germinal center reactions supported by Tfh cells (e.g., IL-4 and IL-21), driving differentiation into antibody-secreting plasma cells and memory B cells. Together, these coordinated adaptive immune responses may provide complementary protection against viral variants arising from viral drift that partially evade neutralizing antibodies.

Circular RNA-based vaccine platforms have recently gained attention as an alternative to linear mRNA vaccines, driven by their enhanced molecular stability and distinct translational features [12]. In the context of prophylactic vaccination, where durable antigen availability and effective immune priming are critical (Figure 2) [22], circRNA offers several mechanistic advantages that may translate into improved vaccine performance.

### 2.1. Translational Characteristics and Prolonged Antigen Expression

Unlike linear mRNA, circRNA relies on cap-independent translation mechanisms, most commonly mediated by internal ribosome entry sites (IRESs) [23]. Multiple studies have demonstrated that circRNA vaccines can support sustained antigen expression compared with linear RNA-based approaches (e.g., the median half-life is at least 2.5 times longer than the linear counterpart in mammalian cells) [24], with translational efficiency being strongly influenced by the choice of IRES and components such as untranslated region (UTR) sequences [25,26,27]. For example, a head-to-head comparison between circRNA and self-amplifying RNA (SAM) SARS-CoV-2 vaccines reported that the circRNA format provided efficient protection in mice and showed improved stability at 4 °C relative to SAM, supporting its suitability for settings where RNA stability is a practical constraint [28]. In addition to SARS-CoV-2, circRNA vaccine concepts have also been extended to RSV, including platforms built on group IIC self-splicing introns to express engineered RSV pre-F antigens and confer in vivo protection [29].

Notably, the choice of IRES and regulatory elements such as UTR has been shown to critically influence translation efficiency [23], suggesting that IRES activity in antigen-presenting cells (APCs), such as dendritic cells, represents an important but underexplored design parameter for circRNA vaccines. For example, viral IRESs can be classified into distinct groups (Groups I to IV); each group requires different initiation factors (IFs) or IRES-trans-acting factors (ITAFs) for efficient translation [30,31]. Consequently, IRES-driven translation would be influenced by cell-type-specific expression profiles of these factors.

Sustained antigen availability during prophylactic vaccination promotes germinal center (GC) reactions by enhancing antigen retention within lymph nodes and supporting GC B cell and T follicular helper (Tfh) cell responses, ultimately facilitating affinity maturation and improved antibody quality [22,32]. Consistent with the importance of sustained antigen availability for GC maintenance, several circRNA vaccine studies report more robust and durable humoral immune responses relative to linear RNA controls [24,33], although direct head-to-head comparisons remain limited.

### 2.2. Innate Immune Activation and Adjuvant-like Effects

In addition to serving as a template for antigen expression, circRNA vaccines may also influence immune responses through innate immune activation, as RNA molecules and their delivery systems can be sensed by pattern-recognition receptors (PRRs), including endosomal Toll-like receptors and cytosolic RNA sensors, leading to the induction of type I interferons and pro-inflammatory cytokines [34]. Some reports suggest that circRNA formulations can induce stronger adaptive immune responses, including enhanced T cell immunity, compared with unmodified linear RNA [24,27,35]. For example, Amaya et al. demonstrated that a circRNA can function as a potent vaccine adjuvant, promoting myeloid and dendritic cell activation in draining lymph nodes and driving strong antigen-specific CD8^+^ T cell responses in vivo [27]. Consistent with this, circRNA vaccines targeting SARS-CoV-2 and its variants have been reported to induce strong antigen-specific CD4^+^ and CD8^+^ T cell responses together with durable humoral immunity in preclinical models [24].

Previously, in vitro binding assays demonstrated that linear and circRNA exhibit distinct receptor-binding preferences, with toll-like receptor 3 (TLR3), retinoic acid-inducible gene I (RIG-I), and melanoma differentiation-associated protein 5 (MDA5) preferentially associating with linear RNA, whereas protein kinase R (PKR), NF90, adenosine deaminase acting on RNA 1 (ADAR1)-p150, and 2′-5′-oligoadenylate synthetase 1 (OAS1) show preferential binding to circRNA [36]. However, further studies will be required to determine how these differential binding patterns translate into functional differences in innate immune signaling in physiological settings, which may also depend on RNA production and purification conditions [17].

Importantly, the observed immunological effects likely reflect not only intrinsic properties of circRNA, such as enhanced molecular stability, but may also be influenced by factors related to RNA production and purification. In this context, residual impurities, such as double-stranded RNA by-products arising from IVT, may contribute adjuvant-like effects that enhance antigen presentation and immune priming [34].

Collectively, these observations point to a broader methodological issue whereby variations in RNA manufacturing, purification, and quality control may substantially affect immunogenicity and complicate direct comparisons between circRNA and linear RNA vaccines. Consequently, interpretation of immune outcomes should take into account the characteristics of the linear RNA control, including its modification status, purity, and formulation.

### 2.3. Humoral Immunity, Neutralizing Antibodies, and ADE Considerations

From a safety perspective, antibody quality is as important as antibody quantity. In some viral infections, non-neutralizing or suboptimal antibodies can contribute to antibody-dependent enhancement (ADE), a phenomenon that has been well documented for flaviviruses such as dengue and Zika viruses, where cross-reactive antibodies with insufficient neutralizing potency can exacerbate infection [37,38]. Although ADE in humans is influenced by multiple factors, including pre-existing immunity, antibody concentration, and Fc receptor engagement, antibody maturation remains a key component shaping protective versus pathogenic antibody responses [37].

By supporting sustained antigen expression and prolonged GC reactions, circRNA vaccines may favor the generation of high-affinity neutralizing antibodies, potentially reducing the risk of ADE. In this regard, the intrinsic stability conferred by the circular RNA structure may contribute to extended antigen availability, which is known to promote affinity maturation during GC responses [22,32]. Nevertheless, the extent to which circRNA-based vaccination influences ADE risk in humans remains to be fully established and likely depends on both antigen design and immunization context, such as the route of administration, dosing regimen, and the underlying immune environment.

Consistent with these considerations, several circRNA vaccine studies have reported robust induction of neutralizing antibodies. For example, circRNA vaccines encoding the receptor-binding domain (RBD) antigens of the SARS-CoV-2 spike protein have been shown to elicit neutralizing antibodies against SARS-CoV-2 and its emerging variants [24]. Beyond RBD-only designs, a circRNA vaccine prototype expressing a variant-informed, prefusion-stabilized spike trimer (VFLIP-X) elicited neutralizing activity across multiple SARS-CoV-2 variants for several weeks after boosting, together with a more balanced Th1/Th2 response profile [39]. In a related context, a circRNA vaccine encoding a dimeric Zika virus envelope domain III fused to an IgG1 Fc fragment, in combination with the nonstructural protein NS1, induced enhanced GC responses and higher titers of neutralizing antibodies while avoiding detectable dengue virus-associated ADE in mouse models [33]. Notably, while neutralizing antibodies primarily target dominant B cell epitopes that are susceptible to viral drift [40], CD8^+^ T cell responses represent an important component of antiviral protection. CircRNA vaccination has been shown to elicit antigen-specific T cell responses, including CD8^+^ T cells [24], and its enhanced molecular stability may contribute to sustained antigen availability. By targeting relatively conserved internal viral antigens, these T cells may provide a complementary layer of defense against variants that partially evade neutralizing antibodies [40].

### 2.4. Multivalent and Next-Generation Vaccine Designs

CircRNA platforms have also been applied to multivalent vaccine designs, including bivalent [28,41], trivalent [42], and tetravalent formulations [41]. In a bivalent setting, a circRNA vaccine simultaneously targeting porcine epidemic diarrhea virus (PEDV) and transmissible gastroenteritis virus (TGEV) was developed using circRNAs encoding the PEDV S1 and TGEV S1 antigens, respectively [41]. Another bivalent circRNA vaccine example was also demonstrated to protect mice against SARS-CoV-2 variants of concern [28]. In a representative trivalent study, a circRNA influenza vaccine encoding neuraminidase (NA) antigens from H1N1, H3N2, and influenza B viruses induced balanced antibody responses against all three NA components and conferred broad protection against heterologous viral challenge in mice [42]. More recently, a tetravalent circRNA vaccine for complex viral pathogens such as monkeypox virus (MPXV) was reported [43]. A circRNA vaccine cocktail encoding four MPXV surface antigens (A29L, A35R, B6R, and M1R), representing both extracellular enveloped virus and intracellular mature virus forms, induced strong neutralizing antibody and T cell responses and provided superior protection against vaccinia virus challenge compared with individual monovalent vaccines [43]. Collectively, these studies demonstrate that multivalent circRNA vaccines can elicit both robust humoral and cellular immune responses, supporting their potential to provide broad protection against complex viral pathogens.

While the bivalent formulation elicited neutralizing antibodies against both PEDV and TGEV viruses, unequal immunogenicity and antigenic competition were observed when the two circRNAs were administered at equivalent doses, necessitating dose optimization and antigen engineering to restore balanced humoral responses [41]. Therefore, the occurrence of antigenic competition in certain bivalent circRNA vaccine studies underscores the importance of rational antigen design, expression balance, and vaccination strategy when advancing multivalent circRNA platforms toward clinical translation.

As an alternative strategy to address the limitations of simultaneous multivalent antigen delivery, the sequential vaccination approach has been evaluated in the context of circRNA vaccines. In the bivalent PEDV/TGEV circRNA study [41], defined prime–boost regimens combining a circRNA–lipid nanoparticle (LNP) vaccine with a commercially available inactivated vaccine (IAV) were compared, including homologous and heterologous schedules. While all regimens induced antigen-specific antibody responses against PS1 and TS1, vaccination schedules incorporating circRNA tended to skew immune responses toward a more Th1-biased profile relative to IAV–IAV. Notably, immune outcomes differed depending on the order of administration, with the IAV–circRNA sequence eliciting higher neutralizing antibody titers against TGEV than the circRNA–IAV schedule, whereas neutralization against PEDV was comparable across regimens. These findings suggest that immune responses can be modulated by the order and combination of vaccine modalities, supporting rational prime–boost scheduling as a complementary strategy for circRNA vaccine optimization [41].

### 2.5. Delivery Strategies and Lymph Node Targeting

Effective engagement of secondary lymphoid organs, particularly draining lymph nodes, is a critical determinant of vaccine efficacy [22,32]. Where lymph node responses have been directly evaluated, circRNA vaccination has been associated with antigen expression and immune activation in lymph nodes draining the site of immunization, together with enhanced GC responses and neutralizing antibody production [27,35,44]. These findings are consistent with established vaccine immunology, in which efficient antigen availability and immune cell priming within lymph nodes support affinity maturation and durable humoral immunity [32]. Consistent with the importance of lymphoid access, a formulation-driven strategy optimized LNP parameters for circRNA encapsulation and in vivo delivery, and intramuscular administration resulted in substantial accumulation in draining lymph nodes together with rapid dendritic cell maturation, supporting the feasibility of engineering LNP composition to bias circRNA vaccine activity toward lymphoid immune priming [45].

Building on these observations, additional delivery strategies have been designed to further enhance lymph node accumulation and thereby amplify circRNA vaccine immunogenicity [35]. In this study, mannose-modified lipid nanoparticles have been shown to increase circRNA delivery to draining lymph nodes, promote dendritic cell maturation, and expand GC B cells, Tfh cells, and long-lived plasma cell populations [35]. In addition, antibody-mediated targeting strategies, such as the use of anti-DEC-205 antibodies, which were chemically conjugated to LNP to direct circRNA-encoded antigens to dendritic cells, have been explored, supporting the feasibility of antibody-guided immune cell engagement [44]. Notably, in the same study, the circRNA platform was further combined with a coordinated immunomodulatory strategy, for example, by incorporating chemokine (C-X-C motif) ligand 13 (CXCL13) to reshape the lymph node cytokine milieu and strengthen GC reactions [44]. Collectively, these observations highlight lymph node access and the coordinated tuning of antigen delivery and the lymph node microenvironment as key design parameters for optimizing circRNA vaccine immunogenicity.

## 3. Circular RNA for Cancer Vaccine

**Figure 3:**
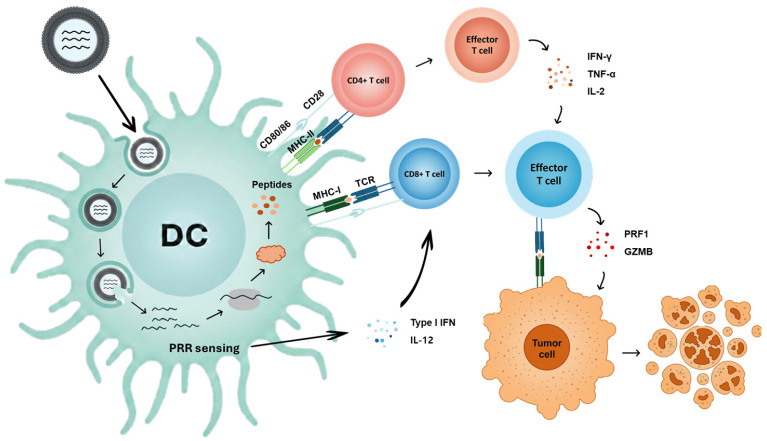
Cancer mRNA–LNP vaccine-induced antitumor T cell immunity. Following administration, mRNA–LNPs are taken up by antigen-presenting cells, including dendritic cells (DCs). As in the prophylactic setting, the mRNA cargo can be recognized by PRRs, where innate immune sensing can induce inflammatory cues (e.g., type I interferons and IL-12) that support T cell priming. In parallel, the delivered mRNA is translated into tumor-associated antigens or neoantigens, which are processed and presented on MHC class I and II molecules for T cell receptor (TCR) recognition, together with co-stimulatory signals (e.g., CD80/86-CD28), to prime CD8^+^ and CD4^+^ T cells. CD4^+^ T cell responses often skew toward a Th1-like phenotype (IFN-γ, TNF-α, IL-2), providing helper signals that promote the expansion and functionality of cytotoxic CD8^+^ T lymphocytes (CTLs). Activated CTLs recognize tumor cells presenting cognate peptide-MHC class I complexes and mediate tumor-cell killing through effector mechanisms such as perforin (PRF1) and granzyme B (GZMB).

Circular RNA-based vaccine platforms are increasingly being explored for cancer immunotherapy, where therapeutic efficacy depends on inducing robust cellular immunity, particularly antigen-specific CD8^+^ T cell responses (Figure 3) [25,26]. Unlike prophylactic vaccination, therapeutic cancer vaccination must contend with central/peripheral tolerance to self-derived tumor antigens, immunosuppressive features of the tumor microenvironment (e.g., Treg (regulatory T cell)- and myeloid-driven suppression), and ongoing antigenic evolution and heterogeneity that enable immune escape [5]. In this context, translatable circRNAs have been evaluated as stable antigen-expression templates, and emerging studies further suggest that immunogenicity and antitumor activity can be tuned through formulation choices and rational combination regimens, as demonstrated in the representative examples discussed below.

### 3.1. Antigen Expression and T Cell-Centric Immunity

Several preclinical studies have demonstrated that translatable circRNA vaccines encoding model antigens, tumor neoantigens, or tumor-associated antigens can elicit robust antigen-specific CD8^+^ T cell responses and measurable antitumor activity in vivo [25,26,27,46]. In a representative PIE-based design, circRNA constructs are paired with viral IRES elements (e.g., CVB3 IRES) to drive cap-independent translation, enabling efficient antigen expression and downstream CD8^+^ T cell priming consistent with MHC class I-restricted antigen presentation (e.g., SIINFEKL/H-2Kb tetramer positive response) [26]. In syngeneic tumor settings spanning distinct immune phenotypes, including an immune-excluded MC38–ovalbumin (OVA) model and an immune-desert orthotopic B16-OVA model, circRNA–LNP vaccination induced antigen-specific T cell responses and tumor growth inhibition that were broadly comparable to nucleoside-modified (m1ψ) linear mRNA–LNP controls [26]. Collectively, these findings underscore the feasibility of circRNA as a translational RNA cancer vaccine platform across multiple tumor models and treatment settings.

Notably, the prolonged translation kinetics associated with circRNA may be particularly useful in therapeutic settings where sustained antigen exposure supports effective T cell priming and expansion. In one representative study, circRNA reporters exhibited more persistent expression than linear mRNA in vitro, consistent with enhanced functional stability over time [26]. In this study, circRNA exhibited an approximately 3-fold higher level of reporter expression than m1ψ-modified linear RNA in NIH3T3 cells at 72 h. In the same work, this property was leveraged in a late-stage orthotopic B16-OVA model, in which vaccination with a circRNA vaccine formulated in lipid nanoparticles was combined with OT-I adoptive T cell transfer. OT-I adoptive T cell transfer refers to the infusion of transgenic CD8^+^ T cells bearing a T cell receptor specific for the OVA-derived SIINFEKL epitope (presented on H-2Kb), which provides a controlled system to track antigen-specific T cell expansion and antitumor activity in vivo. The combination increased OT-I T cell persistence and achieved stronger tumor control than either treatment alone [26]. Building on these findings, subsequent optimization efforts expanded circRNA vaccine designs beyond model antigens to additional therapeutic targets, including B16 neoantigens and human papillomavirus (HPV) E6/E7 constructs, further supporting the adaptability of circRNA-based cancer vaccine platforms for inducing cellular immunity [25]. In these models, the optimized circRNA vaccines elicited antigen-specific interferon (IFN)-γ-producing CD8^+^ T cell responses and showed therapeutic activity, including significant tumor growth inhibition in a B16F10 neoantigen setting and marked tumor reduction in the TC-1 HPV E6/E7 model [25].

### 3.2. Innate Immune Activation and Delivery-Dependent Effects

The immunological outcome of circRNA cancer vaccination is strongly shaped by formulation and delivery, which together determine biodistribution, cellular uptake, and the magnitude and localization of innate immune sensing [4,34,47,48]. In a circRNA–LNP cancer vaccine platform, a design rationale has been described in which circRNA-driven prolonged antigen production supports extended antigen presentation by APCs, whereas highly purified circRNA alone may provide limited innate stimulation and an insufficient proinflammatory milieu for optimal cytotoxic T cell activation. To address this mismatch, the LNP formulation was engineered to include an ionizable lipid capable of inducing proinflammatory cytokine release, thereby coupling sustained antigen expression with an inflammatory context that is consistent with robust CD8^+^ T cell responses and antitumor activity [26]. Similarly, a small circRNA vaccine format has been reported in which compact epitope-encoding circRNAs formulated in lipid nanoparticles enable sustained antigen translation and efficient delivery to antigen-presenting cells in draining lymph nodes, while eliciting innate sensing dominated by RIG-I with relatively low PKR activation [49].

The route of administration can further tune immune compartmentalization. In Amaya et al. [27], mice immunized with circRNA plus ovalbumin protein through subcutaneous, intranasal, or intravenous routes showed distinct distributions of antigen-specific CD8^+^ T cells across the lung, spleen, and draining lymph nodes at both acute and memory time points, and intranasal delivery induced particularly strong lung-focused responses together with lung-resident memory phenotype CD8^+^ T cells expressing CD69 and CD103. In a therapeutic lung metastasis model, an intranasal circRNA–LNP prime–boost regimen achieved antitumor activity comparable to intravenous or intramuscular administration while preferentially confining LNP accumulation and antigen expression to the lung. Intranasal dosing was associated with lower systemic inflammatory signals, including reduced serum IFN-α and attenuated cytokine signatures, as well as milder changes in peripheral blood cell counts. Mechanistically, intranasal vaccination increased antigen-specific CD8^+^ T cells in the lung, including a CD103^+^ tissue-resident memory-like subset, and analyses of antigen-presenting cell subsets implicated lung-resident APCs, such as cDC1 and alveolar macrophages, in supporting local T cell responses [50].

### 3.3. Neoantigen Vaccines and CircRNA-Derived Antigen Space

Beyond canonical tumor-associated antigens (TAAs), circRNA has also been applied to neoantigen-focused therapeutic vaccination, in which tumor-specific epitopes are encoded to elicit cytotoxic CD8^+^ T cell immunity. For example, in a hepatocellular carcinoma (HCC) study, an LNP-formulated circRNA vaccine encoding a PTPN2-derived neoepitope promoted dendritic cell maturation and enhanced DC-driven T cell activation, and it showed therapeutic activity in both subcutaneous and orthotopic murine HCC models. The same platform also provided prophylactic protection when administered before tumor challenge [51].

Beyond mutation-defined neoantigens, non-canonical antigen classes derived from noncoding RNAs have also been explored in circRNA vaccine contexts. For example, a glioblastoma study identified H19-IRP, a protein encoded by lncRNA H19 and presented by MHC class I, and demonstrated that a circRNA vaccine targeting H19-IRP (circH19-vac) elicited cytotoxic T cell responses and inhibited tumor growth in vivo [52].

In parallel, circRNAs themselves have been proposed as an additional, non-canonical antigen reservoir, because back-splice junction-spanning open reading frames can generate peptide sequences absent from the linear proteome [53]. Immunopeptidomic evidence further suggests that selected circRNA-derived peptides can be naturally processed and presented by MHC/HLA, supporting their potential relevance as immunogenic targets [54]. While this emerging concept is not yet central to current circRNA vaccine development, it may broaden the antigen discovery space for personalized cancer vaccines as analytical frameworks for epitope discovery and functional validation advance [54].

### 3.4. Combination Strategies: Adoptive Cell Therapy, In Vivo Cell Engineering, and Chemotherapy

A common direction in RNA-based cancer immunotherapy is to integrate therapeutic vaccination with combination regimens that mitigate tumor-associated immune suppression and amplify T cell-centric activity [5]. In this context, circRNA cancer vaccines have been incorporated into combination-compatible designs, including co-administration with adoptive T cell transfer and a vaccine-responsive chimeric antigen receptor (CAR)-T strategy, supporting their utility as modular boosting components within multimodal regimens [50]. In a related framework, circRNA vaccine boosting using an HER2-encoding circRNA was reported to enhance antitumor immunity in conjunction with in vivo CAR programming (panCAR-VAC concept), further illustrating how vaccination can be integrated into broader cellular immunity-focused strategies [55]. Finally, circRNA-based vaccination platforms can also be integrated with conventional therapies. For example, in a pancreatic cancer model, a circRNA-loaded dendritic cell vaccine combined with low-dose gemcitabine enhanced antitumor immunity, yielding stronger tumor control and improved survival compared with either treatment alone [46].

More recently, attention has expanded toward in vivo CAR reprogramming strategies [56], exemplified by the panCAR-VAC framework [55], that utilize targeted circRNA–LNP delivery to genetically engineer T cells directly within the patient, potentially reducing the logistical complexity associated with conventional ex vivo cell manufacturing [56]. In this setting, the intrinsic molecular stability of circRNA may support more sustained CAR expression compared with linear mRNA templates [55].

Beyond oncology, this in vivo programming paradigm has also been extended to autoimmune indications. Current efforts focus on the targeted delivery of circRNA encoding anti-CD19 CAR constructs to deplete pathogenic B cell populations in autoimmune diseases, with candidates such as SAIL-0804, TI-0032-III, and ORN-252 under evaluation (Table 2). Additional CD19- and BCMA-targeted programs (e.g., ORN-101/145 and ORN-328) are being developed for hematologic malignancies, reflecting the broader application of in vivo CAR engineering strategies. In parallel, circRNA therapeutics are being explored across discovery, preclinical, and early clinical stages for protein replacement and regenerative indications, including RXRG001, CR059, HM2002/2003, and hEpo-circ, alongside vaccine candidates such as TI-0010 and TI-0093 (Table 2). Collectively, these programs illustrate the expanding clinical scope of circRNA platforms across oncologic and non-oncologic settings. When combined with circRNA-based vaccine boosting, such platforms may further enhance the functional persistence of engineered immune cells.

## 4. Considerations, Challenges, and Perspectives

Despite the growing body of preclinical evidence supporting the potential of circRNA-based vaccines in both prophylactic and cancer settings, several limitations and open questions remain that must be addressed to more clearly define their translational value.

### 4.1. Standardization and Interpretability of Comparative Studies

A major limitation across current circRNA vaccine studies is the lack of standardized and appropriately matched linear RNA controls. In many of the studies discussed in this review, circRNA vaccines are compared with linear RNAs that differ in sequence design (e.g., untranslated regions, poly(A) length), chemical modification status, purification level, or LNP formulation, complicating attribution of observed immunological differences to the RNA format itself [21,24,57]. In addition, direct head-to-head comparisons with clinically optimized modified mRNA vaccines are limited [24]. Future studies will require rigorous benchmarking against well-defined linear RNA counterparts to disentangle the effects of circular topology from confounding variables related to RNA design and manufacturing.

### 4.2. Manufacturing, Purification, and Quality Control Considerations

CircRNA vaccine performance may be influenced by production methodology, including in vitro transcription conditions, circularization strategy, and downstream purification. Ribozyme-mediated self-circularization offers practical advantages for scalable manufacturing, yet different implementations (e.g., PIE-based [16,19] or STS-based [21] strategies) introduce distinct sequence architectures and by-product profiles [21,57]. Residual impurities, such as double-stranded RNA species or incompletely circularized/nicked products, may influence innate immune activation and downstream adaptive responses [8]. However, systematic evaluations of how purification strategies and quality control parameters affect immunogenicity remain limited.

CircRNA production, therefore, requires additional process considerations, as nicked RNA species are difficult to fully separate from intact circular molecules, and preservation of covalent circular topology is essential for functional stability [58]. Although techniques such as IP-RP HPLC can facilitate separation at a laboratory scale [21], their scalability and robustness under large-scale manufacturing conditions remain to be established. From a quality control perspective, verification of circular integrity may require complementary orthogonal analytical approaches, including IP-RP HPLC, denaturing PAGE, and RNase R resistance assays to reliably distinguish intact circular RNA from nicked or linear species. Establishing standardized manufacturing and analytical frameworks will be essential for reproducibility and clinical translation.

### 4.3. Translational Control and Immune Cell-Specific Expression

CircRNA translation relies predominantly on cap-independent mechanisms, most commonly mediated by IRESs [59]. IRES-driven translation is strongly dependent on secondary structure, sequence context, and cellular environment [25,30,31]. For example, in a systematic screening of 29 IRES elements, the EV-A IRES exhibited the highest translation efficiency in immune cell lines, showing approximately 1.5-fold higher activity than the reference CVB3 IRES in DC2.4 cells [25]. Structural optimization further increased translation efficiency by approximately 50% following the selective deletion of non-core domains [25]. Preservation of core structural domains was required to maintain functionality, underscoring the importance of structural integrity in determining expression levels [25]. Moreover, differences in IRES performance across DC2.4, THP-1, and HEK293T cells indicate that IRES-mediated expression varies according to cellular context [25,30,31], suggesting that its activity may also be context-dependent in vivo.

Although IRES selection has been shown to critically influence antigen expression [23], systematic screening of translational element performance across vaccine-relevant cell types, including professional antigen-presenting cell populations, remains underexplored. Given the central role of APCs in vaccine efficacy, future circRNA vaccine design may benefit from immune cell-specific optimization of translational elements rather than reliance on broadly active viral IRESs.

### 4.4. Balancing Innate Immune Activation and Adaptive Immunity

Both prophylactic and cancer vaccines require an appropriate level of innate immune activation to support antigen presentation, costimulatory signaling, and germinal center or T cell responses [24,27]. CircRNA vaccines occupy a complex position along this spectrum, as highly purified circRNA exhibits relatively low innate immunogenicity in some settings [21,57]. At the same time, the magnitude of innate stimulation can vary with production, purification (e.g., IP-RP HPLC, SEC, and PAGE), and formulation choices, which together influence the level of residual immunostimulatory by-products and the extent of delivery-dependent adjuvant-like effects [34]. Defining the optimal balance between minimizing excessive innate sensing and preserving sufficient immunostimulation remains a key challenge. Importantly, this balance may differ between prophylactic vaccines, which prioritize durable humoral immunity [24], and cancer vaccines, which require strong cytotoxic T cell responses [27].

### 4.5. Multivalency, Antigen Competition, and Vaccination Strategies

As circRNA platforms are extended to multivalent vaccine designs [42], additional complexities arise. Antigenic competition has been observed in bivalent circRNA vaccines [41], underscoring the need for careful antigen selection, expression balancing, and dosing strategies. Sequential vaccination and heterologous prime–boost regimens have been explored in limited studies but remain under-characterized in the context of circRNA [41]. Rational design of vaccination schedules may therefore be as important as RNA format selection in shaping immune outcomes.

### 4.6. Delivery, Targeting, and Tissue-Specific Immunity

Delivery remains a decisive factor for circRNA vaccine efficacy. Where evaluated, lymph node-targeted or tissue-biased delivery strategies highlight the importance of directing circRNA vaccines to appropriate immune compartments [35,45]. Future progress will likely depend on the co-optimization of circRNA constructs with delivery systems that control biodistribution, cellular uptake, and local immune activation. Such strategies may be particularly relevant for cancer vaccines, where spatially confined immune responses can mitigate systemic inflammatory adverse effects while enhancing therapeutic efficacy [50].

### 4.7. Outlook

**Figure 4:**
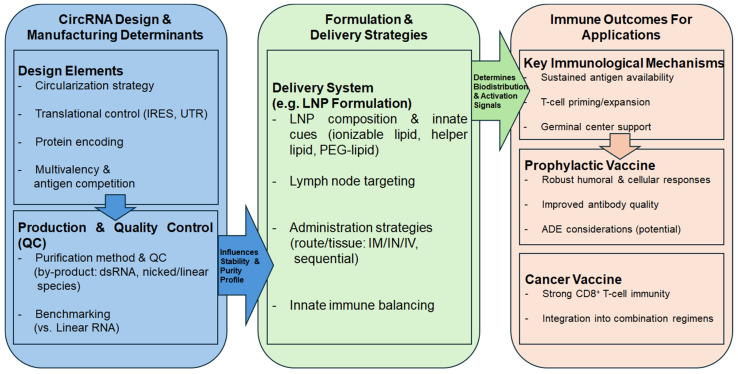
Methodological determinants of circRNA vaccine performance and immune outcomes. This schematic summarizes how linked choices in circRNA design/manufacturing and in formulation/delivery influence downstream immune outcomes. RNA-level variables, including circularization strategy, translation control elements (IRES/UTRs), antigen design, and multivalency, together with production, purification/quality control, and benchmarking against matched linear RNA controls, define the stability and impurity profile of the final circRNA preparation. Formulation parameters (e.g., lipid composition and immunostimulatory cues), lymph node targeting, and administration strategy (route and scheduling) then govern in vivo biodistribution and immunostimulatory signaling. These determinants converge on key immunological mechanisms, including sustained antigen availability, T cell priming/expansion, and germinal center support, thereby shaping prophylactic outcomes (robust humoral/cellular responses and improved antibody quality, with potential implications for ADE) and therapeutic cancer vaccine outcomes (strong antigen-specific CD8^+^ T cell immunity and compatibility with combination regimens).

Collectively, circRNA-based vaccines represent a versatile RNA platform with demonstrated potential across both prophylactic and cancer immunization settings. Their performance is highly context-dependent and shaped by production methodology, translational control, delivery strategy, and immunological objectives (Figure 4).

As circRNA vaccine research progresses, the next step is to translate encouraging preclinical results into practical design principles that support successful clinical translation (Table 2). While mRNA vaccines have established RNA-based vaccination as a viable clinical modality, accumulating evidence suggests that circRNA platforms may offer potential advantages, including improved molecular stability and more sustained antigen expression. To further validate these advantages, future work will benefit from well-controlled head-to-head comparisons with appropriately matched linear RNA controls, deeper mechanistic insight into circRNA translation and innate immune sensing, and systematic co-optimization of RNA design with delivery and vaccination strategies. In parallel, continued efforts to develop scalable and reproducible manufacturing processes, establish standardized quality control frameworks, and benchmark against clinically optimized mRNA platforms will be important for clinical advancement (Table 2). Ultimately, navigating the complex intellectual property landscape, including proprietary IRES elements [60], foundational LNP patent thickets [61], and patents related to circRNA manufacturing methods [62,63], will become a critical determinant of successful commercialization and broad clinical adoption. With these advances, circRNA platforms are well-positioned to expand the RNA vaccine toolkit by enabling more durable antigen expression and tuning immune responses across both prophylactic and therapeutic settings.

## Figures and Tables

**Table 1:** Pros and cons of the different methods for circRNA generation.

Ligation Method	Pros	Cons
Chemical	No intronic scar	Multiple steps Multimer due to DNA splint Unnatural bond
Enzyme	No intronic scar Large GOI	Multiple steps Ligation efficiency
Ribozyme	Simple Large GOI Works in vitro & in vivo	Intronic scar by conventional PIE method Ribozyme as part of transcript By-products after reaction

**Table 2:** Preclinical and clinical trials of circRNA therapeutics or vaccines. Data were compiled from the respective companies’ official announcements, press releases, and ClinicalTrials.gov as of 20 February 2026. Abbreviations: AQP1, aquaporin-1; GLP-1R, glucagon-like peptide-1 receptor; VEGF-A, vascular endothelial growth factor A; BCMA, B cell maturation antigen; EPOR, erythropoietin receptor; R/R, relapsed or refractory; SLE, systemic lupus erythematosus; IND, investigational new drug.

Category	Product	Target	Indication	Phase	Company	Clinical Trial ID
Therapeutics	RXRG001	AQP1	Radiation-induced xerostomia	I/IIa	RiboX	NCT06714253
	SAIL-0804	CD19	Autoimmune diseases	IND-enabling	Sail Bio	-
	CR059	GLP-1R	Type 2 diabetes (T2DM)	I	PegBio	NCT07347080
	TI-0032-III	CD19	R/R Autoimmune diseases	I	Therorna	NCT07413341
	HM2002	VEGF-A	Ischemic heart failure	I	CirCode	NCT06621576
	HM2003	VEGF-A	Thromboangiitis obliterans	Preclinical	CirCode	-
	ORN-101/145	CD19	B cell lymphoma/ Leukemia	Undisclosed	ORNA (* Eli Lilly)	-
	ORN-252	CD19	Refractory SLE/ Autoimmune	I (planned for 2026)	ORNA (Eli Lilly)	-
	ORN-328	BCMA	Multiple myeloma		ORNA (Eli Lilly)	-
	hEpo-circ	EPOR	Renal anemia	Discovery	Sail Bio	-
Vaccines	TI-0010	Spike Protein	COVID-19 and variants	I	Therorna	NCT06205524
	TI-0093	HPV-16 E6/E7	HPV16^+^ solid tumors	I	Therorna	NCT07081984

* The acquisition of Orna Therapeutics (ORNA) by Eli Lilly was publicly announced on 9 February 2026.

## Data Availability

No new data were created or analyzed in this study. Data sharing is not applicable to this article.

## Abbreviations

The following abbreviations are used in this manuscript:

CircRNA	Circular RNA
mRNA	Messenger RNA
COVID-19	Coronavirus disease 2019
SARS-CoV-2	Severe acute respiratory syndrome coronavirus 2
IVT	In vitro transcription
PIE	Permuted intron–exon
SCAP	Split-CVB3 Anabaena-PIE
GOI	Gene of interest
STS	Self-targeting and splicing
IRES	Internal ribosome entry site
UTR	Untranslated region
SAM	Self-amplifying RNA
APC	Antigen-presenting cell
IF	Initiation factor
ITAF	IRES-trans-acting factor
GC	Germinal center
PRR	Pattern-recognition receptor
TLR	Toll-like receptor
RIG-I	Retinoic acid-inducible gene I
MDA5	Melanoma differentiation-associated protein 5
PKR	Protein kinase R
ADAR1	Adenosine deaminase acting on RNA 1
OAS1	2′-5′-oligoadenylate synthetase 1
ADE	Antibody-dependent enhancement
RBD	Receptor-binding domain
PEDV	Porcine epidemic diarrhea virus
TGEV	Transmissible gastroenteritis virus
NA	Neuraminidase
MPXV	Monkeypox virus
LNP	Lipid nanoparticle
IAV	Inactivated vaccine
CXCL13	Chemokine (C-X-C motif) ligand 13
Treg	Regulatory T cell
OVA	Ovalbumin
HPV	Human papillomavirus
IFN	Interferon
TAA	Tumor-associated antigen
HCC	Hepatocellular carcinoma
CAR	Chimeric antigen receptor
EV	Enterovirus
IP-RP HPLC	Ion-pair reversed-phase high-performance liquid chromatography
SEC	Size exclusion chromatography
PAGE	Polyacrylamide gel electrophoresis
